# Comprehensive transcriptomic analysis identifies cholesterol transport pathway as a therapeutic target of porcine epidemic diarrhea coronavirus

**DOI:** 10.1016/j.virusres.2024.199502

**Published:** 2024-11-23

**Authors:** Lilei Lv, Huaye Luo, Min Zhang, Chuntao Wu, Yifeng Jiang, Wu Tong, Guoxin Li, Yanjun Zhou, Yanhua Li, Zhao Wang, Changlong Liu

**Affiliations:** a Shanghai Veterinary Research Institute, Chinese Academy of Agricultural Sciences, Shanghai 200241, PR China; b Office of Academic Research, Dongying Vocational Institute, Dongying 257091, PR China; c School of Laboratory Animal & Shandong Laboratory Animal Center, Shandong First Medical University & Shandong Academy of Medical Sciences, Jinan 250000, PR China; d Department of Laboratory Animal Sciences, School of Basic Medicine, Naval Medical University, Shanghai 200433, PR China; e College of Veterinary Medicine, Yangzhou University, Yangzhou 225009, China; f Jiangsu Co-Innovation Center for the Prevention and Control of Important Animal Infectious Disease and Zoonosis, Yangzhou University, Yangzhou 225009, PR China

**Keywords:** PEDV, Coronavirus, Cholesterol transport, Viral entry, Ezetimibe

## Abstract

Porcine epidemic diarrhea virus (PEDV) is a highly contagious virus that poses a serious threat to the global pig industry. Despite extensive efforts, the mechanism underlying virus entry for PEDV remains elusive. In this study, we first identified PEDV-susceptible and non-susceptible cell lines by using PEDV spike pseudotyped vesicular stomatitis virus. Subsequently, we conducted a comprehensive transcriptomic analysis on these cell lines. Through integrating differential expression gene analysis with weighted gene co-expression network analysis, we identified the key pathways that are correlated with the PEDV entry. Our analysis revealed a strong correlation between cholesterol, sterols, and lipid transport with PEDV entry, suggesting a potential role for cholesterol transport in the PEDV entry. For further investigation, we treated Huh7, Vero and LLC-PK1 cells with a cholesterol transport inhibitor, ezetimibe, and observed a significant inhibition of PEDV entry and subsequent viral replication in these cells. Interestingly, pre-treating Huh7 cells with ezetimibe resulted in an increase in the entry of severe acute respiratory syndrome coronavirus 2 (SARS-CoV-2) and Middle East respiratory syndrome coronavirus (MERS-CoV) pseudoviruses. Moreover, we found that cholesterol could facilitate the entry of PEDV into Huh7 and Vero cells, and this promoting effect can be blocked by ezetimibe. These findings suggest that targeting cholesterol transport specifically inhibits PEDV entry into susceptible cells. Our study offers novel insights into the mechanism of PEDV entry and the development of new therapeutic strategies against this economically important virus.

## Highlights

• PEDV entry is strongly correlated with cholesterol, sterols, and lipid transport.
• The cholesterol transporter NPC1L1 inhibitor, ezetimibe, obstruct the entry of PEDV and subsequent viral replication.
• Pre-treatment of Huh7 cells with ezetimibe promotes the entry of SARS-CoV-2 and MERS-CoV pseudoviruses.

## 1 Introduction

Porcine epidemic diarrhea virus (PEDV), a member of the coronaviridae family, is a highly contagious virus that particularly affects suckling piglets. It induces a severe enteric disease characterized by vomiting, diarrhea, dehydration and high mortality, leading to substantial economic losses in the global swine industry. PEDV is an enveloped, single-stranded, positive-sense RNA virus with a genome of approximately 28 kb in length. PEDV encodes four structural proteins: spike (S), envelope (E), membrane (M),and nucleocapsid (N) (Kocherhans et al., 2001). The PEDV S protein contains two subunits: the receptor-binding subunit S1 and the membrane-fusion subunit S2. During viral entry, the S1 subunit binds to the cell receptors, while the S2 subunit mediates membrane fusion and release viral genome into the cytoplasm(Li, 2016).

The entry of PEDV into host cells is facilitated by several host factors. Previous studies have demonstrated that PEDV uses aminopeptidase N (APN) for host cells entry (Li et al., 2007; Nam and Lee, 2010; Park et al., 2015). However, the role of APN as a receptor for PEDV entry has been questioned (Li et al., 2017; Sun et al., 2018). Recent studies have identified several host factors, including sugars and proteins, that facilitate the entry of PEDV. These factors include sialic acid, heparan sulfate (Huan et al., 2015; Wrapp and McLellan, 2019), DC-SIGN/L-SIGN (Zhao et al., 2021), occluding (Luo et al., 2017), Tfr1 (Zhang et al., 2020) and so on. Following attachment, PEDV utilizes multiple endocytic pathways to enter into host cells, including clathrin-mediated endocytosis, caveolae-mediated endocytosis and lipid-raft mediated endocytosis(Wei et al., 2020). Several therapeutic agents have shown promise in inhibiting PEDV entry. Agents such as 25-hydroxycholesterol (25-HC), RAF265, veratramine, erastin, salinomycin, and niclosamide have been reported to inhibit PEDV replication by blocking viral entry (Chen et al., 2024; Wang et al., 2022, 2023; Yuan et al., 2021; Zhang et al., 2023a, 2019). Understanding the molecular mechanisms underlying PEDV entry and uncovering potential therapeutic targets are critical in the development of specific drugs for PEDV treatment.

PEDV primarily infects villous enterocytes in the small intestine of pigs. In vitro studies have shown that PEDV has the ability to infect cells from diverse species such as humans, monkeys, bats, and rats. Cell lines susceptible to PEDV encompass Vero, Marc-145, LLC-PK1, ST, IPEC-J2, Huh7, HepG2, Hep3B217, SNU387, among others (Lv et al., 2022). Comparing the differential transcriptomic expression between PEDV susceptible and non-susceptible cells can play a pivotal role in identifying key pathways associated with PEDV entry and discovering potential agents for effective PEDV treatment. In this study, we aimed to identify the pathways involved in PEDV entry by analyzing global transcripts in PEDV-susceptible and non-susceptible human cell lines. Using a combination of differential expression gene analysis and weighted gene co-expression network analysis (WGCNA), we discovered a strong association between cholesterol transport and PEDV entry, indicating a potential role of cholesterol transport in the PEDV entry process. We found that a selective inhibitor of cholesterol transport protein NPC1L1, ezetimibe, could inhibit the entry of PEDV and subsequent viral replication in different cell lines. These findings indicate that targeting cholesterol transport could be an effective strategy for controlling PEDV infection.

## 2 Materials and methods

### 2.1 Cells and virus

Vero (CCL-81, ATCC), Huh7 (Procell, Wuhan, China), HepG2 (Procell), Hep3B217 (known as Hep 3B2.1–7, Procell), FU97 (MeiSenCTCC, Hangzhou, China), LLC-PK1 (Procell, Wuhan, China) and HeLa were cultured in DMEM (Hyclone, Shanghai, China) supplemented with 10 % fetal bovine serum (FBS) (Gibco, Shanghai, China) and 1 % penicillin/streptomycin (Gibco). Li-7 (Procell), SNU182 (MeiSenCTCC), SNU387 (MeiSenCTCC), and SNU761 (MeiSenCTCC) were cultured in RIPM 1640 (Hyclone, Shanghai, China) supplemented with 10 % FBS (Gibco) and 1 % penicillin/streptomycin (Gibco). The PEDV G1b strain, PEDV SD (GenBank accession No MZ596343.1), is identified as a trypsin-independent virus strain (Lv et al., 2022). The PEDV G2a strain, PEDV HM (GenBank accession No MZ342899.1) is a trypsin-dependent virus strain (Zhou et al., 2022). Both strains are stored in our laboratory. The recombinant PEDV virus, rPEDV-HM-EGFP, was produced as previously described (Zhou et al., 2022).

### 2.2 Production of PEDV pseudovirus rVSV-ΔG-EGFP-PEDV-S

In order to generate the cell line for packaging PEDV pseudovirus rVSV-ΔG-EGFP-PEDV-S, the human codon optimized full-length spike (S) gene of the PEDV SD was synthesized (Saiheng Biotech, Shanghai, China) and inserted into pLV-EF1a-IRES-Hygro (#85,134, Addgene) vector. The recombinant plasmid pLV-EF1a-PEDV-S-IRES-Hygro with helper plasmids pSPAX2 and pMD2.G were co-transferred into HEK-293T to package lentivirus as previously described (Liu et al., 2018). Huh7 cells were infected with the recombinant lentivirus at an MOI of 5. At 48 h post-infection (hpi), hygromycin (500 μg/mL) was added to cell culture medium and refreshed every 2-3 days for 2 weeks. A positive monoclonal cell line that highly expressed PEDV S protein was obtained by limiting dilution of surviving cells into 96-well plates. Subsequently, the Huh7-PEDV-S-Hygro monoclonal cell line was infected with rVSV-ΔG-EGFP-G (#VSVT, Vector Builder, Guangzhou, China) pseudovirus at an MOI of 5. Following 2 h incubation, the supernatant was removed, and the cells were washed three times with PBS and cultured in fresh DMEM supplemented with 2 % FBS. The supernatant from infected cells was collected at 48 hpi, clarified by 0.45 μm filters, aliquoted, and stored at −80 °C. To eliminate the residue of rVSV-ΔG-EGFP-G, the rVSV-ΔG-EGFP-PEDV-S pseudovirus was purified three rounds on Huh7-PEDV-S-Hygro monoclonal cell line.

### 2.3 Infection of different cell lines with rVSV-ΔG-EGFP-PEDV-S and PEDV SD strain

Huh7, HepG2, Hep3B217, SNU387, FU97, SNU761, SNU182, HeLa, and Li-7 cells were plated in 6-well plates and incubated with rVSV-ΔG-EGFP-PEDV-S (0.05 MOI) or PEDV SD strain (0.1 MOI) for 2 h. Then, the supernatant containing virus was removed. Cells were washed three times with PBS and cultured in fresh DMEM supplemented with 2 % FBS. For rVSV-ΔG-EGFP-PEDV-S infection, the EGFP-positive cells were quantified by flow cytometry analysis at 12 hpi. For PEDV SD infection, the virus titers in supernatant were measured and expressed as tissue culture infective dose 50 % per milliliter (TCID_50_/mL) at 12 and 24 hpi.

### 2.4 TCID_50_ assay

Vero cells were seeded in 96-well plates until reaching 100 % confluence. The viruses were diluted in a ten-fold gradient, and each dilution was inoculated in eight replicates with a volume of 100 mL. Each sample was repeated in three independent groups. The cells were cultured for another five days, during which cytopathic lesions were observed. Virus titers were calculated using the Reed-Muench method and expressed as TCID_50_/mL.

### 2.5 Cell viability assay

The cytotoxicity of ezetimibe in Huh7 and Vero cells was assessed using a CCK-8 kit according to the manufacturer's protocol. Cells were seeded into 96-well plates at a density of 5000 cells per well and incubated for 24 h. Subsequently, the cells were treated with different concentrations of ezetimibe. At 2 h or 24 h, 10 µL of the CCK-8 reagent was added into each well and incubated for 4 h at 37 °C. Measure the absorbance at 450 nm using the microplate reader (Synergy, USA).

### 2.6 SARS-CoV-2 and MERS-CoV pseudoviruses rVSV-∆G-EGFP-SARS-S and rVSV-∆G-EGFP-MERS-S package

To package rVSV-∆G-EGFP-SARS-S and rVSV-∆G-EGFP-MERS-S pseudoviruses, approximately 3 × 10^6^ HEK-293T cells were seeded in a T75 flask and transfected with 24 µg of pCAGGS-SARS-CoV-2-S or MERS-CoV-S plasmid (a gift from Prof. Rong Zhang, Fudan University) using the calcium phosphate method. The cells were incubated at 37 °C with 5 % CO_2_ for 18 h. Then the cells were infected with rVSV-∆G-EGFP-G at an MOI of 5. Following 2 h incubation, the virus was removed, and the cells were washed three times with PBS. Subsequently, 15 mL of DMEM containing 2 % FBS was added to the flask. The supernatants were collected at 48 hpi, centrifuged, filtered, aliquoted, and stored at −80 °C.

### 2.7 Drug treatment and viral infection

Vero, Huh7, or LLC-PK1 cells were seeded in 6-well plates until reaching 90 % confluence. Different doses of ezetimibe (Yuanye, Shanghai, China) or 25-Hydroxycholesterol (MedChemExpress, USA) were added in Vero, Huh7 or LLC-PK1 before or during infection with pseudoviruses, PEDV SD or PEDV HM. The fluorescent cells were observed and photographed at 12 hpi by fluorescence microscope (Zeiss, Germany) and the percentage of fluorescent cells was analyzed by flow cytometer (ACEA, USA). The supernatants containing virus were measured and expressed by TCID_50_/mL.

### 2.8 Cholesterol pre-treatment and pseudovirus infection

Huh7 or Vero cells were pre-treated with different concentrations of water-soluble cholesterol (Sigma-Aldrich, C4951–30MG) for 2 h. Then, the inoculum was removed, and the cells were inoculated with rVSV-ΔG-EGFP-PEDV-S pseudovirus (0.1 MOI) for another 1 h. The number of EGFP-positive cells was examined at 12 hpi using a fluorescence microscope and a flow cytometer.

### 2.9 Flow cytometry analysis

Flow cytometry was used to determine the proportion of pseudovirus-infected cells. Briefly, virus-infected cells were rinsed with PBS, treated with trypsin and resuspended in 400 μL of fluorescence-activated cell sorting (FACS) buffer (PBS containing 2.5 mM EDTA and 2 % FBS). The EGFP-positive cells were determined using a flow cytometer. The acquisition was set at 20,000 events per sample. The data were analyzed using flowJo software (V10, Ashland, OR, USA).

### 2.10 Western blot

The detailed procedure was performed as previously described (Zhang et al., 2023b). The primary antibodies used included GAPDH (1:1000, cat#: 5174, CST) and PEDV-N (1:1000, cat#: YY151229). HRP-conjugated secondary antibodies for rabbit IgG (1:5000, cat#: SA00001–2, Proteintech) and mouse IgG (1:5000, cat#: SA00001–1, Proteintech) were used.

### 2.11 RNA-seq and data analysis

Total RNAs were extracted from the cell lines using the RNeasy Mini Kit (Qiagen, China) following the manufacturer's instruction. The extracted total RNAs were then sent to Saiheng Biotech (Shanghai, China) for sequencing. Briefly, the quality and quantity of the RNAs were assessed using a Bioanalyzer 2100 (Agilent Technologies, China). Only RNA samples with an RNA integrity number (RIN) greater than 9.0 were used for further analysis. The RNA-seq libraries were prepared and sequenced on an Illumina Novaseq 6000. The raw reads were processed and quality controlled using FastQC. Afterwards, the clean reads were mapped to the human reference genome (GRCh38/hg38) using the HISAT2 aligner. The read counts for each gene were calculated using HTSeq, and differential expression analysis was performed using the DESeq2 package in R. All data analysis was conducted in a Mac OS environment with a custom script. The differentially expressed genes (DEGs) were identified based on a false discovery rate (FDR) < 0.01 and a log2 fold change greater than 1 or less than −1. The DEGs were further subjected to functional annotation and pathway analysis for a comprehensive understanding of the underlying biological processes.

### 2.12 Construction of weighted gene co-expression network

To explore the correlation of modules with PEDV entry, we used the average percentage of pseudovirus infection in different cell lines as a PEDV entry factor. Weighted gene co-expression network analysis (WGCNA) package was used to construct a co-expression network and analyze the correlation of modules with PEDV entry according to the previously described protocol (Langfelder and Horvath, 2008). Transcriptomic data from all samples were filtered to remove genes with an FPKM (Fragments Per Kilobase Million) < 1 in all samples. We utilized the one-step function blockwiseModules to construct the co-expression network and identify the modules. This method enabled us to identify modules of co-expressed genes and examine their association with PEDV entry. We then determined an appropriate soft-thresholding power using the pickSoftThreshold function, which facilitated the construction of a weighted adjacency matrix. Hierarchical clustering was performed to group genes based on their topological overlap, resulting in the identification of distinct gene modules. To ensure robustness, we applied a dynamic tree-cutting algorithm with a minimum cluster size of 50 and a merging threshold function of 0.25. Subsequently, we calculated module eigengenes to summarize the expression patterns within each module. Finally, we investigated the correlation between module eigengenes and PEDV entry to uncover potential modules associated with this viral entry process.

### 2.13 Pathway enrichment analysis

Enrichment pathway analysis or gene set enrichment analysis (GSEA) was conducted using the R/Bioconductor package clusterProfiler to identify enriched biological pathways in the selected modules or differentially expressed genes. The analysis was performed based on the Gene Ontology (GO) databases. The enrichGO function was utilized to identify the enriched pathways, and the p-values were adjusted for multiple testing using the Benjamini-Hochberg method. The enriched pathways were visualized using the R enrichplot package. The gseGo function was employed to perform GSEA for all gene expression data. To identify hub genes, the frequency of genes in the enrichment pathways was calculated, and the genes with high frequency were considered as hub genes.

### 2.14 Statistical analysis

The data analysis was performed using R software (version 4.1.0) or GraphPad Prism (version 9, GraphPad, CA, USA). The data are expressed as the mean ± standard deviation (SD) of at least three replicates. The significance of the results was evaluated by performing an unpaired Student's *t*-test and calculating the corresponding P-values. A significance level of < 0.05 (*), < 0.01 (**), and < 0.001 (***) was used to determine the statistical significance.

## 3 Results

### 3.1 Susceptibility of different cell lines to the PEDV pseudovirus rVSV-ΔG-EGFP-PEDV-S

To examine the potential entry factor for PEDV, we utilized a vesicular stomatitis virus (VSV) based pseudovirus rVSV-ΔG-EGFP-PEDV-S, which was packaged with the PEDV spike protein. The G gene of VSV was replaced with the EGFP reporter gene (Luo et al., 2023). This approach allowed us to identify susceptible and non-susceptible cell lines for PEDV infection. We tested a panel of human cell lines, including Huh7, Hep3B217, HepG2, SNU387, FU97, HeLa, SNU182, SNU761, and Li7. Among these, Huh7, Hep3B217, HepG2, SNU387, SNU182, SNU761, and Li7 are hepatocellular carcinoma. Our previous study indicated that Huh7, Hep3B217, HepG2, and SNU387 cell lines were permissive to PEDV infection, whereas the SNU182, SNU761, and Li7 cell lines were non-susceptible (Lv et al., 2022). Additionally, we selected the FU97 and HeLa cell lines for our study. These two cell lines were chosen due to their derivation from different tissue types and may provide additional insights into the mechanisms of PEDV entry.

**Fig. 1:**
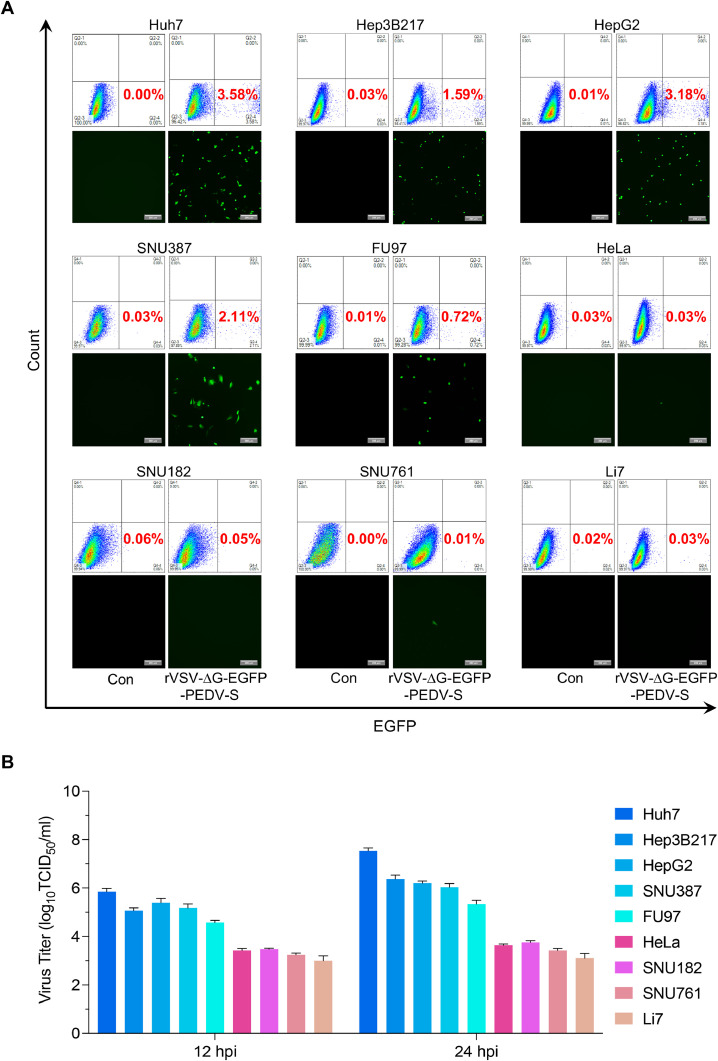
Susceptibility of different cell lines to the PEDV pseudovirus. (A) Different types of cells were infected with 0.1 MOI rVSV-∆G-EGFP-PEDV-S pseudovirus at 37 °C for 1 h. Samples were collected at 12 hpi, and the EGFP-positive cells were analyzed by flow cytometry or imaged by fluorescent microscopy. The green cells were PEDV-infected EGFP cells. Scale bar: 200 μm. (B) Different types of cells were infected with 0.1 MOI PEDV SD at 37 °C for 1 h. The viral titer in supernatants of the infected cells were measured and expressed by TCID_50_/mL at 12 and 24 hpi. Error bars indicate standard deviation of technical triplicates.

We assessed the susceptibility of the above cell lines to rVSV-ΔG-EGFP-PEDV-S pseudovirus by monitoring the expression of the EGFP gene. Flow cytometry and microscope analysis revealed that Huh7, Hep3B217, HepG2, SNU387, and FU97 cell lines were susceptible, whereas HeLa, SNU182, SNU761, and Li7 cell lines showed non-susceptible to rVSV-ΔG-EGFP-PEDV-S pseudovirus infection (Fig. 1A). Subsequently, we examined the permissiveness of these nine cell lines to PEDV. We infected the cell lines with 0.1 MOI PEDV SD strain and detected viral titers in supernatants at 12 and 24 hpi. The results demonstrated that Huh7, Hep3B217, HepG2, SNU387, and FU97 were permissive to PEDV, whereas HeLa, SNU182, SNU761, and Li7 were non-susceptible to PEDV infection (Fig. 1B). These findings aligned with pseudovirus infection assay conducted on the same cell lines, indicating a strong correlation between pseudovirus entry and permissiveness to PEDV. Thus, the inability of the virus to replicate in non-susceptible cell lines can be attributed to its failure to enter these cells.

### 3.2 Transcriptome analysis of PEDV susceptible and non-susceptible cell lines

**Fig. 2:**
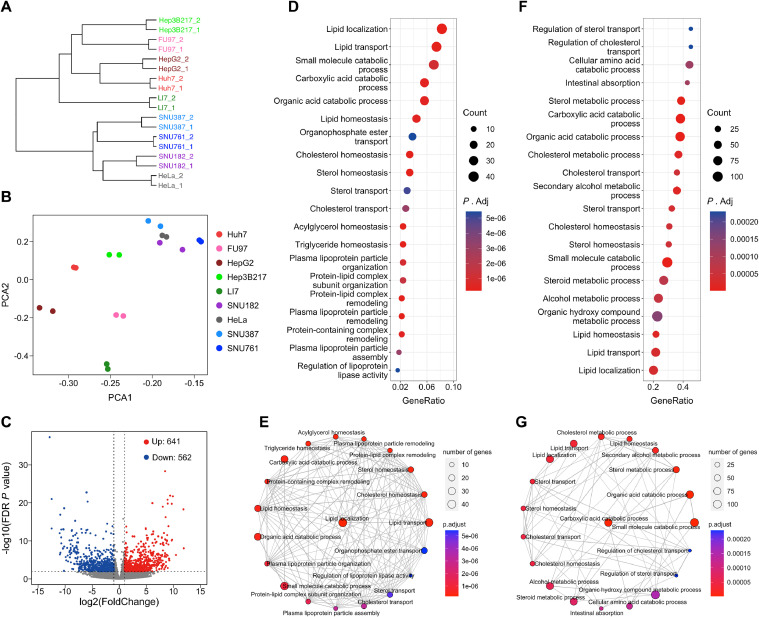
Transcriptome analysis of PEDV susceptible and non-susceptible cell lines. (A) The dendrogram shows the hierarchal clustering of global gene expression between PEDV susceptible and non-susceptible cell lines. The Pearson correlation was used to generate the distance matrix. (B) PCA analysis was performed on the FPKM expression matrix of all samples. (C) The transcriptomes of PEDV susceptible cell lines were compared with and PEDV non-susceptible cells. Volcano plots depict the differential gene expression for each cell line (red and blue as linear fold change of 2 and FDR *P* < 0.01). (D) GO biological process (BP) enrichment analysis of down-regulated differentially expressed genes. The vertical axis shows the top 20 functional classification, and the horizontal axis shows the GeneRatio. (E) Enrichment map showing the network of 20 enriched GO BP pathways from over-representation enrichment analysis with edges connecting overlapped gene sets. (F) GSEA analysis of all genes expressed in different cell lines based on GO BP pathways. The vertical axis shows the top 20 functional classification, and the horizontal axis shows the GeneRatio. (G) Enrichment map showing the network of 20 enriched GO BP pathways from GSEA with edges connecting overlapped gene sets.

The absence of entry factor expression in non-susceptible cell lines is likely responsible for PEDV's inability to enter these cells. Therefore, we conducted RNA sequencing (RNA-seq) experiments on the nine cell lines to elucidate the differences in gene expression profiles between PEDV-susceptible and non-susceptible cell lines. We cultured the cell lines and collected two biological replicates per cell line for analysis. Gene expression levels were evaluated, and we observed that samples within each cell line were correlated with each other, as shown in the samples hierarchical cluster plot (Fig. 2A). Principal component analysis (PCA) further demonstrated the distinguishability of samples from different cell lines (Fig. 2B). We used a false discovery rate (FDR) corrected *P* value < 0.01 and |log2(Fold Change)| ≥ 1 to identify differentially expressed genes (DEGs), resulting in the identification of 1203 DEGs, with 562 down-regulated genes and 641 up-regulated genes in the PEDV non-susceptible cell lines compared to the susceptible cell lines (Fig. 2C). Next, we conducted gene ontology (GO) biological processes (BP) enrichment analysis for the 562 down-regulated genes, which were less expressed in PEDV non-susceptible cell lines than in the PEDV-susceptible cell line. We identified 100 enriched biological processes terms (adjusted *P* < 0.01), and the pathway of lipid localization, lipid homeostasis, lipid transport, cholesterol homeostasis, cholesterol transport, sterol homeostasis, sterol transport pathways were the most significantly different (Fig. 2D). Furthermore, we used an enrichment map to visualize the top 20 enriched pathways (Fig. 2E), indicating that the differentially expressed genes were highly enriched in cholesterol homeostasis, lipid homeostasis, and sterol homeostasis. To validate our GO term enrichment analysis results, we performed gene set enrichment analysis (GSEA) to evaluate the global change of the transcriptome between PEDV non-susceptible and susceptible cell lines, based on the GO database. As expected, the results of the GSEA corroborated that the pathways of lipid localization, lipid homeostasis, lipid transport, cholesterol homeostasis, cholesterol transport, sterol homeostasis, and sterol transport were significantly different, which was consistent with the output of the GO terms enrichment analysis (Fig. 2F and 2G).

### 3.3 Identification of key co-expression modules associated with PEDV entry

**Fig. 3:**
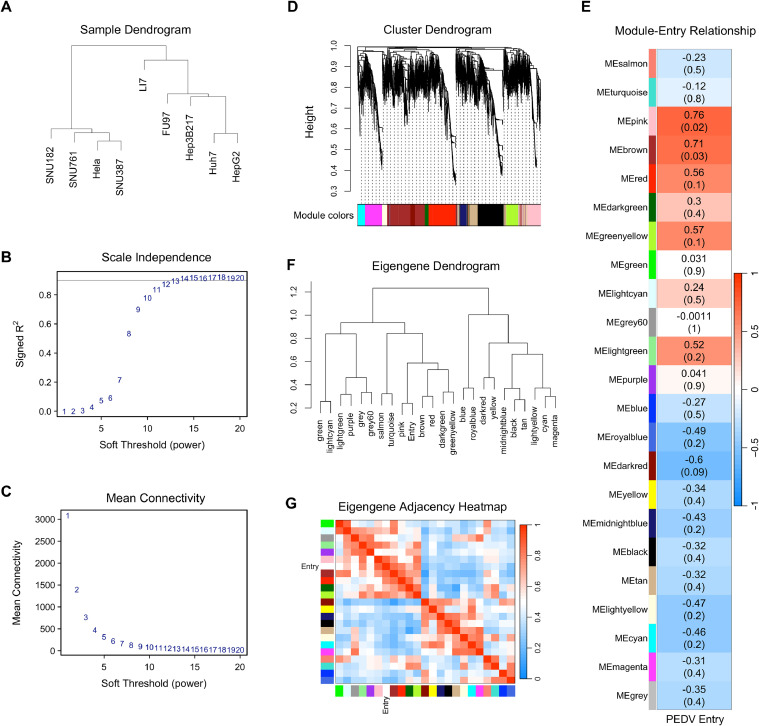
Construction of gene co-expression networks and identification of modules by weighted gene co-expression network analysis. (A) Clustering dendrogram of all cell lines based on their Euclidean distance from RNA-seq FPKM. (B) Analysis of different soft-thresholding power network topology for constructing the scale-free network. The plot shows the scale-free topology model fit, signed R2 (y-axis) as a function of the soft-thresholding power. The grey line shows that the value of the y-axis is 0.9. (C) The plot represents the mean connectivity (y-axis) as a function of the soft-thresholding power. (D) Heatmap depicting the correlation between module eigengenes and PEDV entry. Each row corresponds to a module eigengene and the column to PEDV entry. Each cell contains the corresponding correlation and p-value. The table is color-coded by correlation according to the color legend. (E) Cluster dendrogram of genes in PEDV susceptible and non-susceptible cell lines with dissimilarity based on topological overlap. The different color row below the dendrogram represents module membership clustered by the dynamic tree cut method. (F) The plot shows a hierarchical clustering dendrogram of the eigengenes in which the dissimilarity of eigengenes and PEDV entry. (G) Heatmap plot of the adjacencies of modules and PEDV entry. The colors of columns and row squares represent the adjacency of corresponded modules. Red represents high adjacency, whereas blue represents low adjacency.

To comprehensively investigate the regulatory network associated with PEDV entry, WGCNA was conducted. Before performing, we excluded genes with low expression levels (FPKM< 1). The dendrogram of average FPKM for each gene from duplicated samples were used to identify and remove any outlier samples (Fig. 3A). We then clustered and retained all 9 samples for subsequent analysis. The pickSoftThreshold function in the WGCNA package was used to determine the optimal soft-thresholding powers for calculating the scale-free topology fit index and mean connectivity. We selected a power of 13 as it was the lowest power at which the scale-free topology fit index curve plateaued at a high value 0.9 (Fig. 3B and 3C). Using the dynamic tree cut method, we identified a total of 23 modules based on the strongly interconnected gene clusters that were defined as modules (Fig. 3D). Genes that did not fit into any modules were excluded from further analyses. The 23 modules, along with the correlation and p values are depicted in Fig. 3E. Correlations between PEDV entry and overrepresented modules indicated that the pink module (Correlation coefficient = 0.76, *P* = 0.02) and brown module (Correlation coefficient = 0.71, *P* = 0.03) were positively correlated with the PEDV entry. To further evaluate the interaction among all modules, we calculated eigengene adjacency, which revealed clear distinctions among the modules based on co-expression relationships. The interaction between these co-expressed modules was visualized using the module eigengene dendrogram (Fig. 3F) and eigengene network heatmap (Fig. 3G). Notably, the brown and pink modules showed significant correlation with PEDV entry. The network data from WGCNA was obtained and prepared for further analysis. These findings lay the foundation for identifying potential genes associated with PEDV entry.

### 3.4 Pathway enrichment analysis and hub genes identification

**Fig. 4:**
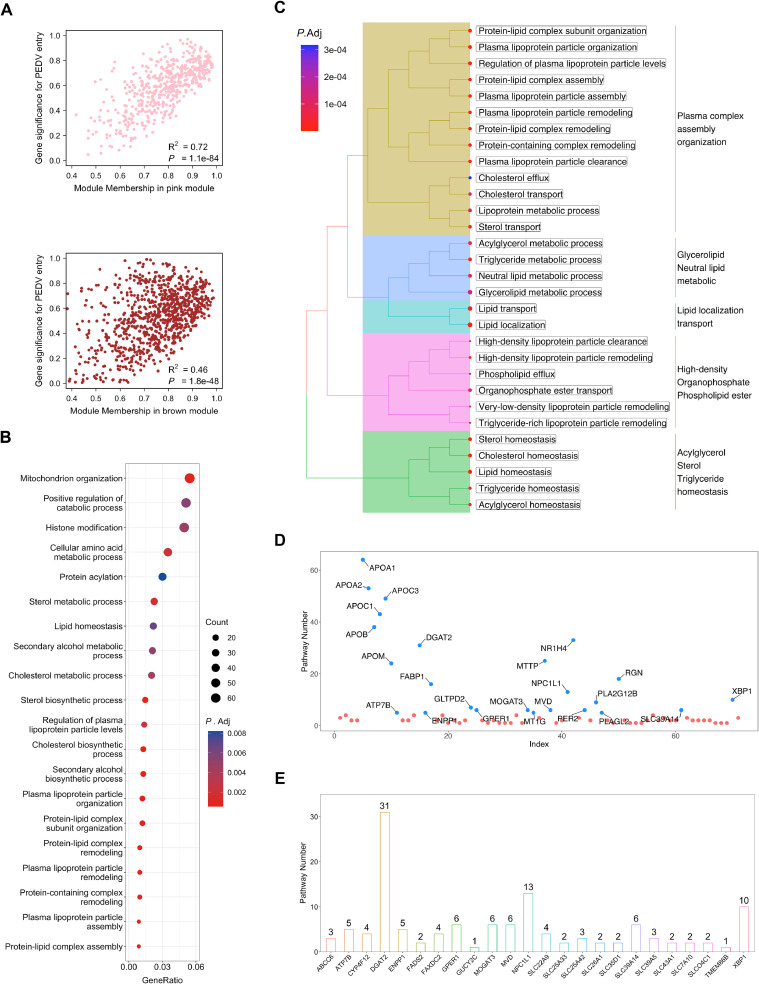
Identification of modules associated with the entry of PEDV. (A) Scatter plot analysis to show the association between module membership (MM) and gene significance (GS) in pink module (top) and brown module (bottom). There is a highly significant correlation between MM and GS in these two modules. (B) GO functional annotation for genes in the pink and brown modules. The x-axis shows the top 20 functional classification, and the y-axis shows the GeneRatio. The size of the nodes is proportional to the number of genes. The colors of the dots represent the *P*-value of each term. (C) GO biological process (BP) enrichment analysis of common genes that were intersected with differentially expressed genes, pink module genes and brown module genes. The enrichment plot shows hierarchical clustering of the top 30 enriched terms. The size of the nodes is proportional to the number of genes. The colors of the dots represent the *P*-value of each term. (D) The plot showed the occurrence frequency of various genes in all the significantly enriched pathways. Blue dots: 5 pathways involved at least. Red dots: <5 pathways involved. (E) The bar plot showed he occurrence frequency of membrane-related genes inall the significantly enriched pathways.

We have identified the pink and brown modules (Fig. 4A), which exhibited a strong positive correlation with PEDV entry, suggesting their potential roles in facilitating viral entry. To better understand the biological functions and processes associated with genes in these modules, we merged all genes from pink and brown modules, resulting in a total of 1000 genes. Subsequently, we performed GO BP enrichment analysis. The analysis revealed that the genes within these modules primarily participate in sterol biosynthetic process, and cholesterol biosynthetic process (Fig. 4B). These processes are crucial for cholesterol metabolism, which may contribute to the successful entry of PEDV. Next, we intersected the genes from pink and brown modules with differentially expressed genes, resulting in 354 genes. We conducted a GO analysis for these genes. As seen in Fig. 4C, these genes are essential for lipid homeostasis, lipid transport, cholesterol homeostasis, cholesterol transport, sterol homeostasis, and sterol transport and so on (Fig. 4C). Further investigation into the specific genes within these modules may provide novel targets for antiviral therapeutics against PEDV infection. To identify the hub genes involved in these pathways, we analyzed the frequency of each gene across all 80 pathways. Among the 71 genes present in these pathways (Fig. 4D), 24 were annotated as membrane localization genes (Fig. 4E) based on the annotation from the human protein atlas (https://www.proteinatlas.org). To further refine our candidate gene list with a specific focus on genes related to the plasma membrane, we identified Niemann-Pick C1-Like 1 (NPC1L1) as the hub gene (Fig. 4E), which is a well-known plasma membrane gene that has been extensively studied in the context of cholesterol transport.

### 3.5 NPC1L1 inhibitor ezetimibe inhibits PEDV entry

**Fig. 5:**
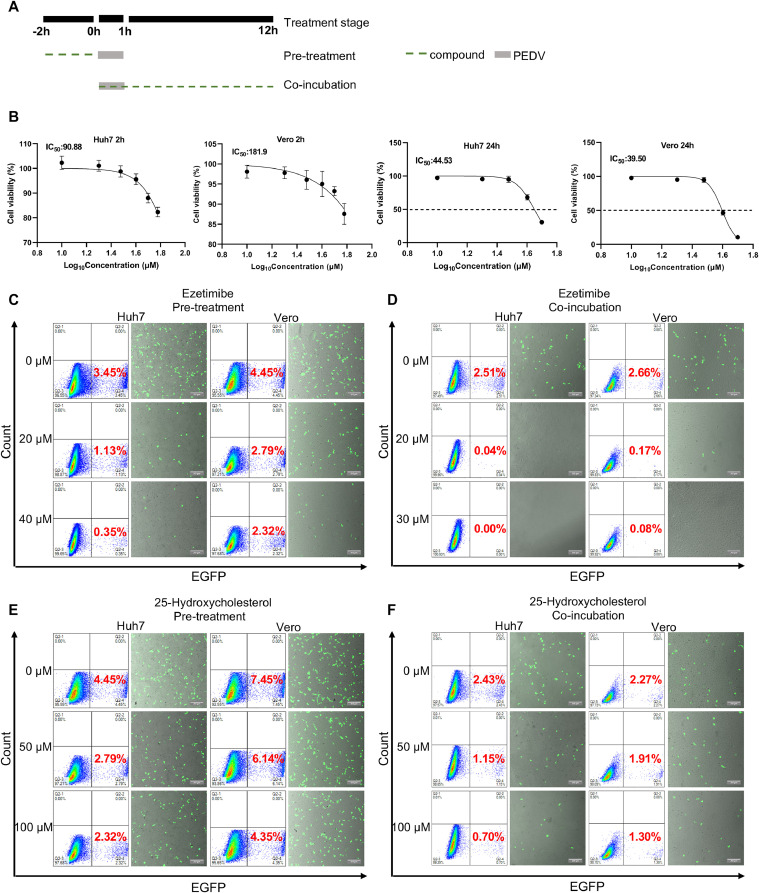
NPC1L1 inhibitor ezetimibe inhibits PEDV entry. (A) Overview of the schedule of ezetimibe or 25-HC treatment and PEDV infection assays. The gray box refers to the PEDV infection period and the green line refers to the ezetimibe or 25-HC treatment. (B) Huh7 or Vero cells were treated with 0 μM, 10 μM, 20 μM, 30 μM, 40 μM, and 50 μM ezetimibe for 2 h or 24 h. Then CCK-8 was added and incubated for another 4 h. The OD_450_ value was measured. Huh7 and Vero cells were pre-treated with different doses of ezetimibe (C) or 25-HC (E) for 2 h. Then, the inoculum was revmoved and infected with 0.1 MOI rVSV-∆G-EGFP-PEDV-S pseudovirus for another 1 h. Huh7 and Vero cells were co-incubated with different doses of ezetimibe (D) or 25-HC (F) and 0.1 MOI rVSV-∆G-EGFP-PEDV-S pseudovirus for 2 h. Then, removed the inoculum and added fresh DMEM containing corresponding doses of ezetimibe or 25-HC. Samples were collected at 12 hpi and the number of EGFP-positive cells was analyzed by flow cytometry or imaged by fluorescent microscopy. The green cells were PEDV-infected EGFP cells, and the background cells were shot with white light. Scale bar: 200 μm.

We speculated that NPC1L1 may involve in the entry process of PEDV. This hypothesis is supported by our pathway enrichment analysis suggesting that cholesterol metabolism, cholesterol transport, and cholesterol homeostasis are closely linked to PEDV entry. Ezetimibe, an effective and selective NPC1L1 inhibitor, can attach to the NPC1L1 protein, thereby blocking intestinal cholesterol absorption (Garcia-Calvo et al., 2005). Considering the potential involvement of NPC1L1 in PEDV entry, we hypothesized that ezetimibe could obstruct PEDV pseudovirus infection. To validate this, Huh7 and Vero cells were treated with ezetimibe or a positive control, 25-HC, known for its antiviral activity against PEDV by blocking viral entry (Zhang et al., 2022, 2019), before or during infection with 0.1 MOI rVSV-ΔG-EGFP-PEDV-S pseudovirus (Fig. 5A). The IC_50_ of ezetimibe on Huh7 and Vero cells were determined through a cell viability assay (Fig. 5B) and the maximum dose of 25-HC was determined to be 100 μM based on the previous research(Zhang et al., 2022). After 12 hpi, samples were collected, and the number of EGFP-positive cells was analyzed by flow cytometry or fluorescent microscopy. The results showed that the number of EGFP-positive cells was significantly reduced in both ezetimibe-treated Huh7 and Vero cells (Fig. 5C and 5D). As expected, 25-HC also exhibited a restriction on PEDV entry (Fig. 5E and 5F). Collectively, these findings indicate that ezetimibe effectively inhibits the entry of PEDV.

### 3.6 NPC1L1 inhibitor ezetimibe inhibits PEDV replication

**Fig. 6:**
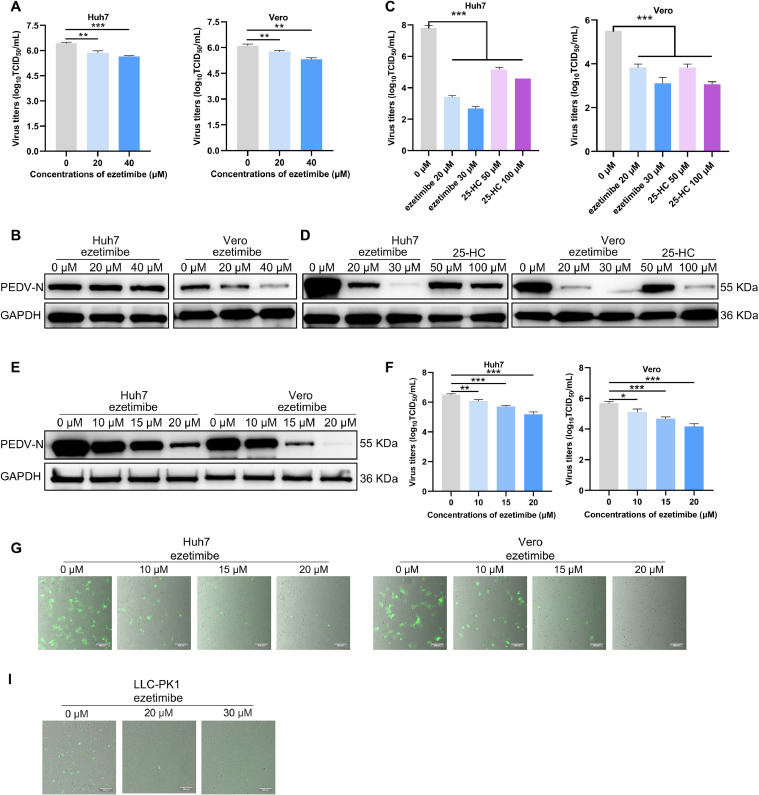
NPC1L1 inhibitor ezetimibe inhibits PEDV replication. Huh7 (A) and Vero (B) cells were pre-treated with different doses of ezetimibe for 2 h. Then, removed the inoculum and infected with 0.1 MOI PEDV SD for another 1 h. The viral supernatants were collected at 12 hpi. Huh7 (C) and Vero (D) cells were co-incubated with ezetimibe or 25-HC and 0.1 MOI PEDV SD for 1 h. Then, removed the inoculum and added fresh DMEM containing corresponding doses of ezetimibe or 25-HC. The viral supernatants were collected at 24 hpi. The infectious PEDV in supernatants were titrated and expressed by TCID_50_ assay, and the expression level of PEDV N protein was measured by Western blot assay (E). Huh7 and Vero cells were co-incubated with different doses of ezetimibe, 0.1 MOI PEDV HM and 0.25 % trypsin (5 μg/mL for Huh7 cells and 10 μg/mL for Vero cells) for 1 h, and then removed the inoculum and added fresh DMEM containing corresponding concentrations of ezetimibe and 0.25 % trypsin. The viral RNA copy levels in supernatants of the infected cells were quantified by RT-qPCR at 24 hpi (G), the expression level of PEDV N protein was measured by Western blot (F). (H) Huh7 and Vero cells were co-incubated with different doses of ezetimibe, 0.1 MOI rPEDV HM-EGFP and 5 ug/ml of trypsin for 24 h and the EGFP fluorescent cells were imaged by fluorescent microscopy. The green cells were PEDV-infected EGFP cells, and the background cells were shot with white light. (I) LLC-PK1 cells were infected with rPEDV-HM-EGFP at an MOI of 0.1, supplemented with various doses of ezetimibe and 5 µg/ml of trypsin for 2 h. Following the infection, the virus was removed, and the cells were cultured in fresh medium containing different concentrations of ezetimibe for an additional 12 h. The EGFP fluorescence in the cells was subsequently analyzed using fluorescence microscopy. The green cells represented PEDV-infected EGFP cells, while the non-infected cells were captured under bright field. Scale bar: 200 μm. Error bars represent standard deviations of technical triplicates. *P < 0.05, **P < 0.01, ***P < 0.001, *n* = 3, technical repeat.

To further validate the inhibitory effect of ezetimibe on PEDV infection, Huh7 and Vero cells were treated with different doses of ezetimibe or 25-HC before or during 0.1 MOI PEDV SD infection. Subsequently, the viral titer in supernatants was measured. The results demonstrated that both pre-treatment or co-incubation with ezetimibe or 25-HC could significantly inhibit PEDV replication in a dose-dependent manner in Huh7 and Vero cells (Fig. 6A-D). Subsequently, the corresponding expression levels of PEDV N protein in both Huh7 and Vero cells were assessed by Western blot analysis. Consistent with the results of virus titers, ezetimibe treatment significantly restricts the intracellular PEDV N protein levels (Fig. 6E).

Two genotypes, G1 and G2, have been identified based on the genetic diversification of the S gene for PEDV. Strains within the G1 and G2 subgroups have different characteristics. The PEDV SD strain is classified under the G1 subgroup. To examine the impact of ezetimibe on the replication of the PEDV G2 strain, we treated Huh7 and Vero cells with different doses of ezetimibe during infection with 0.1 MOI PEDV G2 strain HM. Our analysis revealed that ezetimibe significantly inhibited the replication of PEDV HM, as demonstrated by the intracellular viral N protein levels and copy numbers of the viral genome (Fig. 6F and 6G). Additionally, we infected Huh7 and Vero cells with 0.1 MOI rPEDV HM-EGFP, a recombinant PEDV expressing EGFP derived from the PEDV G2 strain, during ezetimibe treatment. In accordance with the results from PEDV HM strain, a reduction in EGFP signal was observed in ezetimibe treatment cells in a dose-dependent manner (Fig. 6H). Furthermore, the impact of ezetimibe treatment was evaluated on the porcine cell line LLC-PK1 using the recombinant virus rPEDV-HM-EGFP. Consistent with the results in Huh7 and Vero cells, a reduction in EGFP signal was observed in the ezetimibe treated LLC-PK1 cells in a dose-dependent manner (Fig. 6I). Taken together, these findings underscore the potential of ezetimibe as a therapeutic agent for combating PEDV infection.

### 3.7 Ezetimibe pre-treatment increases the entry of SARS-CoV-2 and mers-cov

**Fig. 7:**
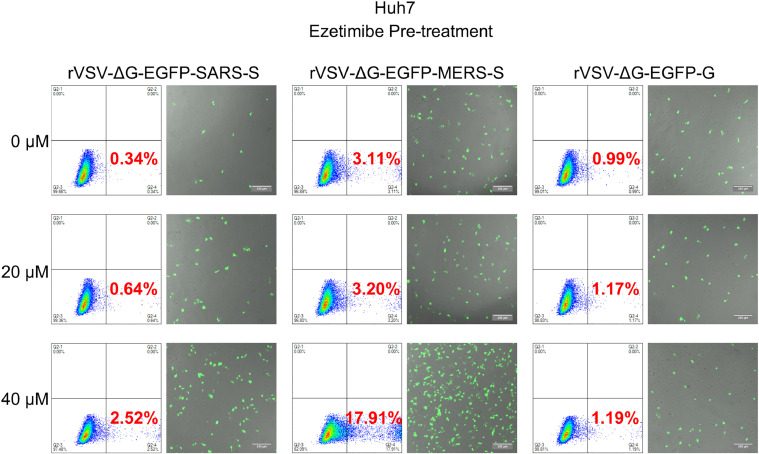
Ezetimibe pre-treatment increases the entry of SARS-CoV-2 and MERS-CoV. Huh7 cells were pre-treated with 0 μM, 20 μM, or 40 μM ezetimibe for 2 h. Then, removed the inoculum and infected with 0.1 MOI rVSV-ΔG-EGFP-SARS, rVSV-ΔG-EGFP-MERS, or rVSV-ΔG-EGFP-G pseudoviruses for another 1 h. Samples were collected at 12 hpi and the number of EGFP-positive cells was analyzed by flow cytometry or imaged by fluorescent microscopy. The green cells were PEDV-infected EGFP cells, and the background cells were shot with white light. Scale bar: 200 μm.

Cholesterol plays a crucial role in the entry and replication of various coronaviruses. Cholesterol-rich microdomains, known as lipid rafts, in the host cell membrane serve as platforms for virus binding. Depletion of cholesterol from the host cell membrane using pharmacological agents, such as methyl-β-cyclodextrin (MβCD), has been shown to significantly reduce the entry and replication of SARS-CoV, MERS-CoV and SARS-CoV-2(Palacios-Rapalo et al., 2021). Given the evidence that ezetimibe can inhibit the entry of PEDV, we then verified whether the inhibitory effect of ezetimibe on PEDV is specific. To test this hypothesis, we packaged VSV-based pseudoviruses carrying the spike proteins of SARS-CoV-2 and MERS-CoV, named rVSV-ΔG-EGFP-SARS-S and rVSV-ΔG-EGFP-MERS-S, respectively. Then, Huh7 cells were pre-treated with different doses of ezetimibe and infected with 0.1 MOI rVSV-ΔG-EGFP-SARS-S, rVSV-ΔG-EGFP-MERS-S pseudoviruses as well as the control pseudovirus rVSV-ΔG-EGFP-G. Subsequently, samples were assessed by analyzing the EGFP-positive cells using fluorescence microscopy and flow cytometry. Interestingly, we found that ezetimibe pre-treatment significantly increased the infection of rVSV-ΔG-EGFP-SARS-S and rVSV-ΔG-EGFP-MERS-S pseudoviruses (Fig. 7). In contrast, the efficiency of the control rVSV-ΔG-EGFP-G infection was not affected by ezetimibe pre-treatment (Fig. 7). Taken together, these findings imply that ezetimibe specifically inhibits PEDV entry.

### 3.8 Ezetimibe impedes the role of cholesterol in facilitating the entry of PEDV

**Fig. 8:**
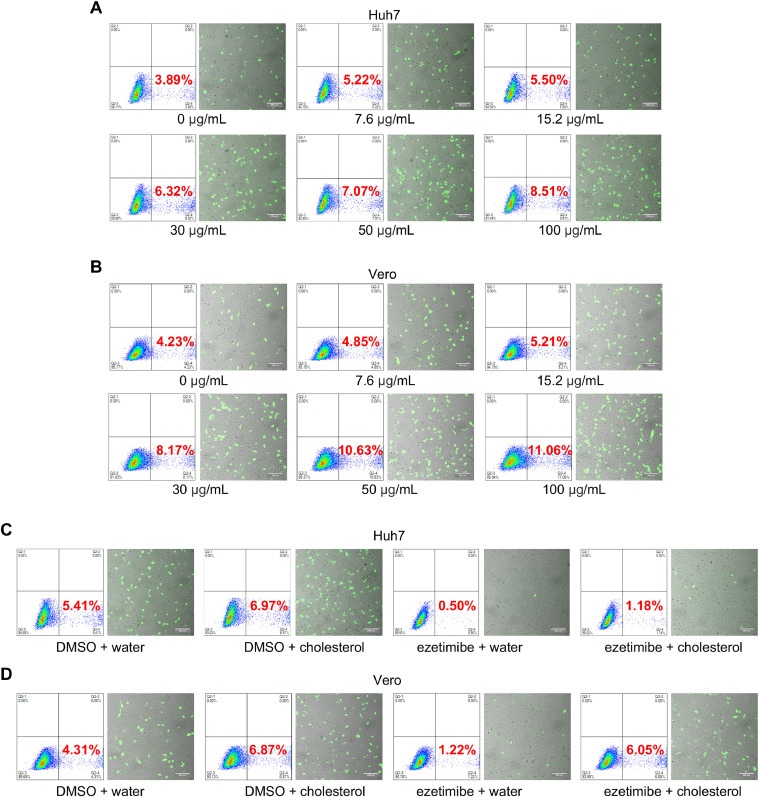
Ezetimibe impedes the role of cholesterol in facilitating the entry of PEDV. Huh7(A) and Vero (B) cells were pre-treated with different concentrations of cholesterol for 2 h. Then, removed the inoculum and infected with 0.1 MOI rVSV-ΔG-EGFP-PEDV-S pseudovirus for another 1 h. Samples were collected at 12 hpi and the number of EGFP-positive cells was analyzed by flow cytometry or imaged by fluorescent microscopy. Huh7 (C) and Vero (D) cells were pre-treated with 40 μM ezetimibe or DMSO for 2 h, and add 30 μg/mL cholesterol or cell culture water for another 2 h. Then, removed the inoculum and infected with 0.1 MOI rVSV-ΔG-EGFP-PEDV-S pseudovirus for 1 h. Samples were collected at 12 hpi and the number of EGFP-positive cells were analyzed by flow cytometry or imaged by fluorescent microscopy. The green cells were PEDV-infected EGFP cells, and the background cells were shot with white light. Scale bar: 200 μm.

It has been reported that cellular cholesterol is required for the entry process of PEDV and porcine deltacoronavirus (Jeon and Lee, 2017, 2018). To confirm the role of cholesterol in PEDV entry, Huh7 and Vero cells were treated with different concentrations of water-soluble cholesterol prior to infection with rVSV-ΔG-EGFP-PEDV-S pseudovirus. The infection efficiency was subsequently assessed by analyzing the EGFP-positive cells using fluorescence microscopy and flow cytometry. Our results demonstrated a significant increase in the number of EGFP-positive cells in both Huh7 and Vero cells pre-treated with cholesterol compared to the untreated control in a dose-dependent manner (Fig. 8A and 8B). These findings strongly suggest that cholesterol plays a crucial role in facilitating the entry of PEDV into host cells.

Our transcriptome analysis shows that PEDV entry is strongly correlated with cholesterol transport. To investigate whether cholesterol, which facilitates PEDV entry, depends on the cholesterol transport pathway, Huh7 and Vero cells were treated with ezetimibe before and during cholesterol treatment, followed by infection with rVSV-ΔG-EGFP-PEDV-S pseudovirus. The EGFP-positive cells were examined by flow cytometry and fluorescent microscopy. The number of EGFP-positive cells decreased after ezetimibe treatment compared to untreated cells in both cholesterol-treated Huh7 and Vero cells (Fig. 8C and 8D). These results indicate that the facilitation of PEDV entry by cholesterol is dependent on the cholesterol transport pathway.

## 4 Discussion

PEDV is a highly contagious virus that affects pigs of all ages. The virus is known to spread rapidly and cause high mortality in newborn piglets, leading to significant economic losses in the pig industry. There are no specific drugs or effective vaccines for the treatment of PEDV (Lin et al., 2022). The entry of PEDV into cells is mediated by the PEDV spike protein binding to host cell receptors. However, the details of PEDV entry mechanism remain unclear (Li et al., 2022). PEDV mainly infects porcine small intestine epithelial cells. Besides, PEDV could infect cells from diverse species such as humans, monkeys, bats, and rats, suggesting that the entry process of PEDV may be conserved across species. In this study, we screen pathways and molecules related to PEDV entry by comparing PEDV human susceptible and non-susceptible cell lines. Compared to porcine cell lines, human cell lines provide a broader range of options for our analysis. Additionally, the annotation of the human genome is more comprehensive and accurate compared to that of the porcine genome. However, the use of human cells to screen for host pathways and molecules associated with porcine virus entry has certain limitations. For example, PEDV may enter cells of different species through a variety of mechanisms. Using VSV pseudotypes displaying PEDV spike protein, we screened a panel of nine human cell lines and identified five susceptible cell lines (Huh7, Hep3B217, HepG2, SNU387, FU97) and four non-susceptible cell lines (HeLa, SNU182, SNU761, Li7). The susceptibility of these lines to pseudovirus entry strongly correlated with their permissiveness to wildtype PEDV infection. This indicates viral entry as the key determinant of cell permissiveness for these cell lines. Pseudoviruses are invaluable tools for studying specific stages of the viral life cycle due to their ability to safely simulate certain virus-host interactions without the inherent safety risks of handling live, pathogenic viruses. However, it is important to note that results from pseudovirus experiments may not always accurately reflect real viral infections. This discrepancy arises because pseudoviruses may lack specific components present in wild-type, or naturally occurring, viruses. These missing components, such as accessory proteins, play crucial roles in host cell interaction and immune evasion, which are essential for understanding genuine viral behavior. In the case of coronaviruses, these accessory components can significantly influence processes such as viral entry, replication, and disease progression. Given these considerations, our findings from pseudovirus studies need to be further validated using the actual PEDV virus.

Our differentially expressed gene analysis combining WGCNA revealed downregulation of cholesterol metabolism related pathways in PEDV non-susceptible lines compared to susceptible lines, suggesting that cholesterol metabolism related pathways play an essential role in PEDV entry. Cholesterol is an essential component of the cell membrane and plays a crucial role in the formation and stability of lipid rafts. Lipid rafts are specialized microdomains within the cell membrane that are enriched in cholesterol and sphingolipids. These rafts are highly dynamic and serve as platforms for various cellular processes, including signal transduction, membrane trafficking, and protein sorting. Furthermore, cholesterol in lipid rafts is involved in the regulation of membrane trafficking. It affects the formation and stability of lipid rafts, which in turn influence the sorting and trafficking of proteins within the cell. It has been reported that cholesterol-rich lipid rafts serve as platforms for virus entry. These rafts contain various receptors and proteins that are essential for the virus to infect cells (Ripa et al., 2021). Moreover, cholesterol plays an important role in the internalization of clathrin-, caveolae-coated vesicles (Ripa et al., 2021; Subtil et al., 1999). PEDV enters cells through clathrin-, caveolae-, and lipid raft-mediated endocytosis (Wei et al., 2020). Cholesterol has been shown to play a critical role in entry and replication of coronaviruses, including SARS-CoV, MERS-CoV (Palacios-Rapalo et al., 2021; Wang et al., 2020; Zang et al., 2020), porcine deltacoronavirus (Jeon and Lee, 2018; Maj et al., 2021), TGEV(Yin et al., 2010) and PEDV(Jeon and Lee, 2017; Wei et al., 2020; Zhang et al., 2019) . Depletion of membrane cholesterol using methyl-β-cyclodextrin or interference with cholesterol biosynthetic pathway by statins or 25-HC significantly inhibits coronaviral entry and replication. Our transcriptomic data aligns with these earlier findings and provides additional bioinformatic evidence demonstrating the importance of cholesterol-associated pathways in determining PEDV entry. These findings highlight the complexity of PEDV entry mechanisms. Identifying key host molecules associated with PEDV entry may reveal novel targets for antiviral drugs and inform the development of more effective vaccines.

We identified the NPC1L1 gene as a top hub plasma membrane gene positively correlated with PEDV entry. The NPC1L1 gene is primarily expressed in the small intestine, where it plays a crucial role in cholesterol absorption. The main function of NPC1L1 is to facilitate the uptake of dietary cholesterol from the intestinal lumen into the enterocytes, which are the cells lining the small intestine. It acts as a cholesterol transporter, allowing cholesterol to enter the enterocytes for further processing and absorption (Jia et al., 2011). Previous studies have shown NPC1L1 to be exploited by other viruses like hepatitis C virus and norovirus for cell entry or replication (Sainz et al., 2012). Our study expands this paradigm and suggests NPC1L1 as an attractive candidate interacting with PEDV. Using ezetimibe, a selective pharmacological inhibitor of NPC1L1, we demonstrated inhibition of both PEDV pseudovirus infection and PEDV (both G1 and G2 strains) entry and subsequently replication in Huh7 and Vero cells. The antiviral efficacy of ezetimibe was higher compared to 25-HC, an established inhibitor of PEDV entry that acts through the depletion of membrane cholesterol (Zhang et al., 2019). We also confirm this findings in porcine cell line. IPEC-J2 is a widely recognized cell line for studying PEDV infection, being derived from neonatal pig jejunum (Brosnahan and Brown, 2012). While some studies have demonstrated the susceptibility of IPEC-J2 cells to PEDV infection, others have reported inconsistent results (Zhang et al., 2018; Zhao et al., 2014). Based on our own experiments, we encountered challenges with low infection efficiency in IPEC-J2 cells. To overcome these limitations, we opted to utilize LLC-PK1 cells, another porcine cell line that is commonly employed to validate the effects of treatments on PEDV infection. Our results showed that co-incubation with ezetimibe significantly inhibited PEDV infection in LLC-PK1 cells when exposed to the rPEDV HM-EGFP strain, indicating ezetimibe could be a candidate for combating PEDV infection.

Unexpectedly, pre-treatment with ezetimibe enhances the entry of SARS-CoV-2 and MERS-CoV pseudoviruses. One potential explanation for this finding may be the alteration of the localization of ACE2 and DPP4, known entry receptors for SARS-CoV-2 and MERS-CoV, by ezetimibe (Raj et al., 2014; Starr et al., 2020). Another possibility is that ezetimibe induces alterations in cholesterol-rich microdomains within the host cell membrane, potentially aiding in the attachment, fusion, or internalization of viral spike proteins. Further characterization of the precise mechanisms through which ezetimibe boosts the entry of these pseudoviruses is crucial, as it could significantly impact our comprehension of coronavirus entry and potentially guide the development of innovative therapeutic approaches.

Our omics and pharmacological data implicate the role of NPC1L1 in PEDV entry. Ezetimibe treatment could obstruct the translocation of NPC1L1 from the cell membrane to the cytoplasm. However, overexpression of NPC1L1 in PEDV non-susceptible cells has a minimal effect on PEDV replication (Data not shown), suggesting that the process of PEDV entry is independent on NPC1L1.

The process of enveloped viral entry can be broken down into five discrete steps, including attachment, signaling, endocytosis, penetration, and uncoating. PEDV membrane fusion mainly occurrs in late endosomes and lysosomes, relying on low pH environment and proteolytic cleavage to release viral genomes into the cytoplasm(Wei et al., 2020). Our results show that ezetimibe treatment inhibits PEDV entry but its specific target protein NPC1L1 is not involved, suggesting that ezetimibe treatment does not influence the attachment process of PEDV, but rather the endocytosis, penetration and uncoating phases by disrupting cellular cholesterol homeostasis. One potential explanation may be the disruption of PEDV-dependent clathrin-, caveolae-, or lipid raft-mediated endocytosis processes, which are cholesterol-dependent. Another possibility is that ezetimibe prevents the fusion between viral and endosomal-lysosomal membranes, resulting in the inability of uncoating and the viral genome is eventually degraded in lysosomes. Cholesterol is known to play a significant role in the life cycle of various viruses, influencing viral entry, assembly, and release, and could similarly affect PEDV. Ezetimibe-treated induces intracellular cholesterol imbalance, which may affect the formation of ER-derived replication organelles and then restrict PEDV replication. Future studies could explore these aspects by examining different PEDV strains and the specific influence of cholesterol on PEDV infection and replication in vivo. These investigations would provide more comprehensive insights and enhance the applicability of our findings across diverse PEDV variants.

## 5 Conclusion

In conclusion, our study identified key pathways associated with the entry of PEDV into susceptible cells. Through comprehensive transcriptomic analysis and differential gene expression analysis, we found a strong correlation between cholesterol, sterols, and lipid transport with PEDV entry. Through the inhibition of cholesterol transport using ezetimibe, we demonstrated a significant reduction in PEDV entry and subsequent viral replication in Huh7, Vero and LLC-PK1 cells. Interestingly, cholesterol was found to facilitate PEDV entry and could be blocked by ezetimibe, suggesting a potential therapeutic strategy for inhibiting PEDV. Furthermore, our findings highlight the importance of targeting cholesterol transport in combating PEDV and provide valuable insights for the development of new therapeutic approaches against this economically significant virus.

## CRediT authorship contribution statement

**Lilei Lv:** Writing – review & editing, Writing – original draft, Software, Methodology, Investigation, Formal analysis, Data curation. **Huaye Luo:** Methodology, Investigation. **Min Zhang:** Methodology, Investigation. **Chuntao Wu:** Software. **Yifeng Jiang:** Validation, Resources. **Wu Tong:** Validation, Resources. **Guoxin Li:** Validation, Resources. **Yanjun Zhou:** Visualization, Resources. **Yanhua Li:** Visualization, Resources. **Zhao Wang:** Supervision, Project administration, Funding acquisition, Formal analysis, Data curation, Conceptualization. **Changlong Liu:** Writing – review & editing, Writing – original draft, Visualization, Supervision, Software, Project administration, Methodology, Funding acquisition, Formal analysis, Data curation, Conceptualization.

## Declaration of competing interest

The authors declare that they have no known competing financial interests or personal relationships that could have appeared to influence the work reported in this paper.
